# Autoantikörper und die autoreaktive Immunantwort: ACPA sind mehr als nur ACPA

**DOI:** 10.1007/s00393-020-00887-z

**Published:** 2020-09-24

**Authors:** H. U. Scherer

**Affiliations:** Department of Rheumatology, Leiden University Medical Center, P.O. Box 9600, 2300RC Leiden, Niederlande

**Keywords:** Rheumatoide Arthritis, Posttranslationale Modifikation, B‑Zellen, Homocitrulline, Acetyllysin, Rheumatoid arthritis, Posttranslational modification cells, B cells, Homocitrulline, Acetyllysine

## Abstract

Die Immunantwort gegen posttranslational modifizierte Antigene, insbesondere die Entwicklung von Autoantikörpern gerichtet gegen citrullinierte Proteine („anti citrullinated protein antibodies“, [ACPA]), ist ein sehr spezifisches Phänomen der rheumatoiden Arthritis. Bis heute ist unklar, wie es zur Entwicklung dieser Immunantwort kommt und welche Faktoren dazu beitragen, dass aus dieser zunächst asymptomatischen Autoimmunreaktion eine Autoimmunerkrankung entsteht. Analysen zu genetischen Risikofaktoren legen nahe, dass T‑Helfer-Zellen hierbei eine wichtige Rolle zukommt. Unter ihrem Einfluss kommt es zu einer Reifung der citrullinspezifischen B‑Zell-Immunantwort im Vorfeld des Erkrankungsbeginns. Welche Trigger diese Entwicklung stimulieren ist nicht bekannt. Neue Daten zeigen, dass ACPA nicht nur citrullinierte Antigene erkennen. Auch andere Eiweißmodifikationen wie Homocitrullin und Acetyllysin werden spezifisch erkannt. Diese Kreuzreaktivität konnte für verschiedene monoklonale ACPA nachgewiesen werden. Hierdurch erweitert sich das Spektrum der Antigene, durch die ACPA-exprimierende B‑Zellen stimuliert und aktiviert werden können. Auch T‑Zellen, die selbst nicht autoreaktiv sind, sondern Fremdantigene erkennen, treten als mögliche Helfer in den Vordergrund. Die vorliegende Übersichtsarbeit gibt einen Einblick in die Bedeutung dieser neuen Erkenntnisse für das Krankheitsbild der rheumatoiden Arthritis.

## Abstract (en)

The immune response against posttranslationally modified (PTM) antigens, in particular the generation of anti-citrullinated protein antibodies (ACPA), is a very specific hallmark of rheumatoid arthritis. The factors that initiate this immune response and the triggers that stimulate the transition from asymptomatic autoimmunity to autoimmune disease are so far unknown. Genetic risk factors and the maturation of the ACPA response prior to the onset of arthritis indicate an important role for helper T cells in this process. Antigens that trigger this process, however, remain to be defined. Notably, recent data demonstrate that ACPA do not only recognize citrullinated protein antigens. Other posttranslational protein modifications such as homocitrulline and acetyllysine are also recognized. This cross-reactivity towards different PTM antigens was found for various monoclonal ACPA and broadens the spectrum of antigens that can stimulate and activate ACPA-expressing B cells. Also, it suggests that such B cells could receive help from autoreactive but also from non-autoreactive T cells. This review summarizes these recent findings and provides insight into their potential relevance for the disease rheumatoid arthritis.

Antikörper, gerichtet gegen citrullinierte Proteine („anti citrullinated protein antibodies“ [ACPA]), sind hochspezifische Marker der rheumatoiden Arthritis (RA). Der Nachweis von ACPA bei Gelenkbeschwerden ist prädiktiv für den Ausbruch der Erkrankung. Da ACPA oft lange vor dem Auftreten der Arthritis im Serum nachweisbar sind, stellt sich die Frage, wodurch ACPA entstehen, warum sie persistieren und welche Faktoren den Übergang zum Erkrankungsbild der RA induzieren. Neue Erkenntnisse zur Kreuzreaktivität der vermeintlich citrullinspezifischen Antikörper werfen Licht auf diese Frage und suggerieren mögliche Antworten.

## Autoimmunität als physiologischer Bestandteil des Repertoires

Das adaptive Immunsystem besitzt die Fähigkeit, gegen praktisch jegliche erdenkliche und für den Organismus völlig unbekannte (bio)chemische Struktur Antikörper zu entwickeln. Diese Fähigkeit beruht auf einem Repertoire von geschätzt >10^13^ unterschiedlichen Rezeptoren, die durch die zufällige Rekombination von Gensegmenten entstehen und in B‑Lymphozyten als B‑Zell-Rezeptoren (BZR) zur Expression kommen [8]. Zwangsläufig entstehen hierbei auch Rezeptoren, die körpereigene Antigene erkennen und binden. Tatsächlich sind im frühesten Stadium der B‑Zell-Entwicklung ca. 75 % der noch unreifen B‑Zellen autoreaktiv. Viele der autoreaktiven B‑Zellen werden eliminiert („klonale Deletion“), wodurch die Diversität des BZR-Repertoires abnimmt. Ein Teil der BZR erkennt jedoch auch Fremdantigene, die Autoantigenen strukturell ähnlich sind. Häufig ist die Avidität dieser BZR für das Autoantigen gering. Um Schutz durch Diversität zu gewährleisten und „Löcher“ im Repertoire zu verhindern, wird ein gewisses Maß an Autoreaktivität „akzeptiert“. De facto sind ca. 15 % der reifen, naiven B‑Zellen im peripheren Blut autoreaktiv und erkennen Autoantigene, meist mit niedriger Avidität [10]. Somit ist Autoreaktivität also Teil des physiologischen Repertoires naiver B‑Zellen.

## Vorübergehende Autoreaktivität versus pathologische, autoreaktive Immunantwort

Bei ca. 1–2 % der gesunden Bevölkerung können IgG-ACPA im Serum nachgewiesen werden [6, 9]. Die Prävalenz dieser serologischen Befunde bei Gesunden ist allerdings höher als die Prävalenz ACPA-positiver RA (ca. 0,5–0,8 %). Auch Verwandte ersten Grades von RA-Patienten können ACPA entwickeln, allerdings oft nur vorübergehend [7]. Dies zeigt, dass autoreaktive Immunantworten bei Gesunden zwar entstehen, aber nicht zwangsläufig persistieren und dass, zumindest bei der RA, nicht jede Autoantikörperantwort zur Erkrankung führt. Für Letzteres bedarf es weiterer, möglicherweise externer Trigger sowie genetischer Risikofaktoren (Suszeptibilität).

Der Aufbau einer ausgereiften, persistierenden Immunantwort bedarf der Keimzentrumsreaktion, während derer B‑Zellen unter dem Einfluss von CD4^+^-T-Helferzellen („T-Zell-Hilfe“) den Antikörperisotyp wechseln, durch somatische Hypermutation reifen und zu B‑Gedächtniszellen und/oder Antikörper sezernierenden Plasmazellen differenzieren [2]. Die starke Assoziation zwischen Genpolymorphismen der HLA(„human leukocyte antigen“)-Klasse-II-Moleküle und zahlreichen Autoimmunerkrankungen unterstreicht die entscheidende Rolle, die CD4^+^-T-Helferzellen bei der Entwicklung persistierender, autoreaktiver Immunantworten zukommt. Auch die ACPA-Immunantwort zeigt Zeichen der T‑Zell-abhängigen Reifung vor Beginn der Erkrankung [6]. ACPA erkennen zeitnah vor Erkrankungsbeginn eine zunehmende Zahl citrullinierter Antigene („epitope spreading“), die Zahl nachweisbarer ACPA-Isotypen nimmt zu, und auch die Serumtiter steigen. Mit der RA assoziierte HLA-Polymorphismen (bekannt als „shared epitope“ Allele) erhöhen das Risiko für die Entwicklung ACPA-positiver RA, sind jedoch nicht assoziiert mit dem alleinigen Auftreten von ACPA [3]. Es ist daher wahrscheinlich, dass die ACPA-Immunantwort unter dem Einfluss von T‑Helferzellen ausreift, dass diese Reifung dem Krankheitsbild vorausgeht und auch notwendig ist für die Entwicklung der RA (Second-hit-Modell). Diese Entwicklung könnte durchaus über einen längeren Zeitraum erfolgen, während dem ACPA-exprimierende B‑Zellen in wiederholten Keimzentrumsreaktionen T‑Zell-Hilfe erhalten, wodurch die ACPA-Immunantwort schrittweise reift (Multiple-hit-Modell). Letztlich ergibt sich hieraus die Frage, welche Antigene die T‑Zell-Antwort stimulieren, die zur Ausreifung der ACPA-B-Zell-Immunantwort führt und somit zum Übergang von transienter, physiologischer Autoreaktivität zu einer persistierenden, möglicherweise pathogenen Autoimmunreaktion.

## ACPA, ACarP, AAPA, AMPA … Kreuzreaktivität als Basis des Toleranzverlusts

Die meisten gängigen Modelle der B‑Zell‑/T-Zell-Interaktion gehen davon aus, dass beide Zelltypen verschiedene Epitope des gleichen Antigens erkennen. Im Falle eines Autoantigens sind also sowohl B‑ und T‑Zellen autoreaktiv. Schon länger ist bekannt, dass individuelle ACPA verschiedene citrullinierte Antigene erkennen, also mit Blick auf citrullinierte Antigene kreuzreaktiv sind. Die strukturelle Ursache hierfür wurde kürzlich anhand von Kristallstrukturen erkennbar [1]. Die antigenbindende Domäne der citrullinspezifischen Antikörper bildet eine hydrophobe, polare Tasche, durch die Citrullin als Eiweißmodifikation der Aminosäure Arginin gebunden wird. Die benachbarten Aminosäuren im Protein nehmen zwar an der Antigenbindung teil, sind allerdings für die spezifische Bindung des Citrullins von untergeordneter Bedeutung. Dies bedeutet, dass eine ACPA-exprimierende B‑Zelle durch verschiedene citrullinierte Antigene aktiviert werden und somit auch von unterschiedlichen T‑Zellen Hilfe erhalten kann, solange diese eines der citrullinierten Antigene erkennen, mit denen die B‑Zelle kreuzreagiert. Neuere Studien zeigen allerdings, dass die ACPA-Kreuzreaktivität noch sehr viel weiter geht.

Neben ACPA wurden in den letzten Jahren bei RA-Patienten Autoantikörper beschrieben, die andere, posttranslationale Eiweißmodifikationen (PTM) erkennen. Insbesondere Antikörpern gegen carbamylierte und acetylierte Proteine („anti-carbamylated protein antibodies“ [ACarP] bzw. „anti-acetylated protein antibodies“ [AAPA]) kommt hier eine besondere Bedeutung zu. Diese erkennen spezifisch die Aminosäuren Homocitrullin bzw. Acetyllysin, beides strukturell ähnliche Modifikationen der Aminosäure Lysin. Da es sich bei Lysin um eine andere Aminosäure handelt als Arginin, sind carbamylierte und acetylierte Antigene per Definition nicht identisch mit citrullinierten Antigenen und befinden sich an anderer Stelle im jeweiligen Protein. Dennoch konnten Studien mit monoklonalen ACPA zeigen, dass diese vermeintlich citrullinspezifischen Antikörper in hohem Maß kreuzreagieren mit carbamylierten und acetylierten Antigenen [5]. Kristallstrukturen von monoklonalen ACPA mit acetylierten oder carbamylierten Antigenen liegen noch nicht vor. Es ist jedoch nicht unwahrscheinlich, dass beide Modifikationen in vielen Fällen ebenfalls in die Tasche der antigen-bindenden Domäne passen und so weitestgehend unabhängig von den benachbarten Aminosäuren erkannt werden können. Aufgrund der ausgeprägten Kreuzreaktivität werden die autoreaktiven Immunantworten nun zusammengefasst und als AMPA („anti-modified protein antibodies“) bezeichnet.

**Figure 1:**
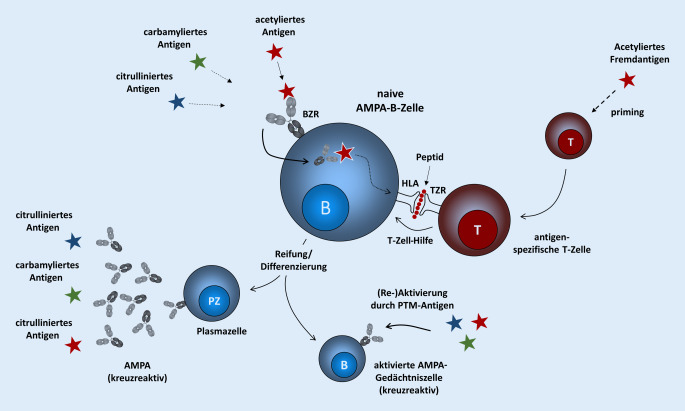

Für die Entstehung und Reifung der autoreaktiven Immunantwort ist die Beobachtung der Kreuzreaktivität hochrelevant, da hierdurch das Spektrum möglicher T‑Zellen, die zur Ausreifung der ACPA- bzw. AMPA-Immunantwort beitragen, wesentlich zunimmt. Die Acetylierung von Proteinen ist ein häufiges Phänomen in Bakterien, wodurch nun auch mikrobielle Proteine als Trigger in den Fokus rücken. Darüber hinaus verfällt die Notwendigkeit, dass sowohl B‑ als auch T‑Zelle autoreaktiv sind. De facto kann eine nichtautoreaktive T‑Zelle, die ein mikrobielles, z. B. acetyliertes Fremdeiweiß erkennt und durch dieses aktiviert wird, einer autoreaktiven, AMPA-exprimierenden B‑Zelle helfen und somit die Kontrollmechanismen der immunologischen Toleranz durchbrechen (Abb. 1).

## Der Bruch immunologischer Toleranz im Mausmodell

Im Mausmodell konnte gezeigt werden, dass immunologische Toleranz durch posttranslationale Modifikation von Proteinen in vivo tatsächlich durchbrochen werden kann [4]. Mäuse, die mit acetyliertem Ovalbumin (einem Fremdeiweiß) immunisiert wurden, entwickelten Antikörper gegen acetyliertes und carbamyliertes Fibrinogen (ebenfalls ein Fremdeiweiß), wohingegen die Immunisierung mit nicht modifiziertem Ovalbumin keine Immunantwort gegen Fibrinogen auslöste. Dies zeigt, dass durch Modifikation eines Proteins eine Immunantwort induziert wird, die auch andere Modifikationen spezifisch erkennt (Kreuzreaktivität), unabhängig von dem zur Immunisierung verwendeten Protein. Auch nach Immunisierung mit carbamyliertem Ovalbumin wurde dieses Phänomen beobachtet. Interessanterweise entwickelten die immunisierten Mäuse aber auch eine Immunantwort gegen acetyliertes und carbamyliertes murines Albumin (ein körpereigenes Protein). Durch die Modifikation des Ovalbumins wurde also nicht nur Kreuzreaktivität induziert, sondern auch eine Autoimmunantwort. Toleranz wurde durchbrochen.

## Ausblick – Prävention der RA?

Die beschriebenen Beobachtungen zeigen, wie Autoimmunität gegen PTM-Proteine entstehen kann, und machen es wahrscheinlich, dass externe (möglicherweise mikrobielle) Trigger die Reifung der AMPA-Immunantwort vorantreiben. Da diese Reifung eng verbunden ist mit der Entwicklung der RA, kommt der Identifizierung dieser Trigger eine wichtige Bedeutung zu. Konzeptuell ist es denkbar, dass dieser Prozess in einem frühen Stadium, d. h. vor der HLA-Klasse-II-assoziierten Reifung der Immunantwort, noch reversibel ist.

## Fazit für die Praxis

- Bei der RA finden sich Autoantikörper gegen verschiedene, posttranslational modifizierte Proteine (AMPA).
- AMPA sind in hohem Maße kreuzreaktiv.
- Die Modifikation eines Proteins alleine ist ausreichend, um immunologische Toleranz zu durchbrechen.
- CD4^+^-T-Helferzellen, die zur Entstehung und Reifung der AMPA-Immunantwort beitragen, müssen selbst nicht zwangsläufig autoreaktiv sein.
- Autoimmunreaktionen können in einem frühen Stadium reversibel sein.
